# Towards synthetic fillers for fair photo lineups: application of generative AI in criminal law proceedings

**DOI:** 10.3389/frai.2026.1834376

**Published:** 2026-07-07

**Authors:** A. Dörsch, R. Nichols, C. Busch, C. Rathgeb

**Affiliations:** da/sec - Biometrics and Security Research Group, Hochschule Darmstadt, Darmstadt, Hesse, Germany

**Keywords:** eyewitness identification, face recognition, generative AI, lineup fairness, photo lineups, synthetic data

## Abstract

A photo lineup is an identification procedure widely used in criminal investigations. It involves presenting a suspect embedded into a set of known-innocent candidates (fillers) to an eyewitness in order to identify the suspect. Compiling fair lineups remains a challenge, particularly when the investigation team has to resort to unsuitable fillers. To support real-world lineups and mitigate the risk of misidentification, we present a practical approach for generating synthetic fillers. By injecting weighted layer-wise noise into a suspects latent vector representation, our approach generates visually distinct fillers while largely preserving demographic characteristics of the suspect. To assess suitability for investigative scenarios, we conducted a large-scale human perception study involving over 450 participants. The results show that the use of synthetically generated fillers leads to balanced identification performance, without making the suspect indistinguishable or stand out. Further experiments show that, compared to previous work, our approach achieves improved preservation of demographic characteristics. Overall, our work contributes to improving fairness in lineups and opens up avenues for supporting criminal investigations through the use of synthetic data.

## 1 Introduction

Eyewitnesses play a crucial role in forensic investigations by contributing to the identification of a suspect. An identification procedure frequently used in the police context is the lineup. In this procedure, an eyewitness is presented with a group of individuals, consisting of a suspect and a set of known-innocent candidates, termed *fillers*. The witness is then asked to identify the culprit in question or state that the alleged culprit is not present. If the witness successfully identifies the person suspected of a crime, this judgment may be considered investigative evidence and made available to law enforcement authorities (U.S. Department of Justice, 1999). Under controlled lineup conditions, eyewitness identification can serve as reliable indicators of accuracy (Wixted et al., 2016).

There are two lineup presentation methods: the sequential lineup, in which lineup candidates are presented to the witness one at a time, and the simultaneous lineup, in which all lineup candidates are presented to the eyewitness at once (Wells and Olson, 2003). In a sequential lineup, the witness makes a binary decision (regarding the class culprit or filler), before viewing the next lineup candidate (Wells and Olson, 2003). This procedure is intended to reduce the risk of relative judgment decisions (Wells et al., 1998), where witnesses tend to mistakenly identify a filler, who appears to be most similar to the suspect in a simultaneous lineup (Wells et al., 2015). However, it was found that advantages of sequential lineups over simultaneous ones further depend on several factors such as lineup fairness (e.g., the lineup arrangement) (Carlson et al., 2008). To reduce the risk of unintentional influences on decision-making, it is recommended that lineups are conducted using a double-blind procedure, i.e., neither the witness nor the (police) attendant knows who the suspect is (Wells et al., 1998).

Although the lineup identification procedure remains consistent in the global scope, the utilized medium formats vary considerably depending on the location of the criminal investigation and are influenced by country-specific policies (Fitzgerald et al., 2018). While countries such as the United States of America and Canada predominantly (but not exclusively) use photo lineups (static mug shots), others such as the United Kingdom and Wales favor video lineups (video sequences of the candidates) (Fitzgerald et al., 2018). Some countries rely on traditional live lineups (where candidates are met in person on site), even experimental results have demonstrated no clear advantage of live lineups over digital medium formats (Rubinova et al., 2021).

Regardless of the medium used, a major challenge in assembling a fair lineup is to select suitable fillers who resemble the suspect without introducing cognitive bias. For example, if the fillers do not resemble the suspect sufficiently, they stand out and increase the risk of misidentification by the witness. At the same time, fillers who appear too similar to the suspect may reduce the likelihood of a correct identification. Since false eyewitness identifications of innocent suspects have contributed to numerous miscarriages of justice in the past, an ideal selection of fillers (e.g., based on matching descriptions and/or appearances) (Tunnicliff and Clark, 2000) is of utmost importance to protect innocent individuals and ensure fair lineups. Recent research conducted by Colloff et al. (2021) found that description-matched fillers (individuals sharing the same demographic characteristics but are visually distinct) lead to fair lineups and improve eyewitness identification performance. Consequently, an ideal filler should largely reflect a suspect's demographic characteristics (e.g., age, gender, ethnicity) while exhibiting sufficient variations in identity cues (facial similarity). However, the reliability of finding ideal fillers may vary depending on a suspect's demographic characteristics. For example, it can be more difficult to find suitable fillers for a suspect from a less represented demographic group. This may potentially lead to biased lineups where the investigation team is reliant on certain fillers that do not meet ideal conditions (e.g., differ significantly in demographic characteristics) due to a shortage of suitable fillers in their available databases.

**Figure 1:**
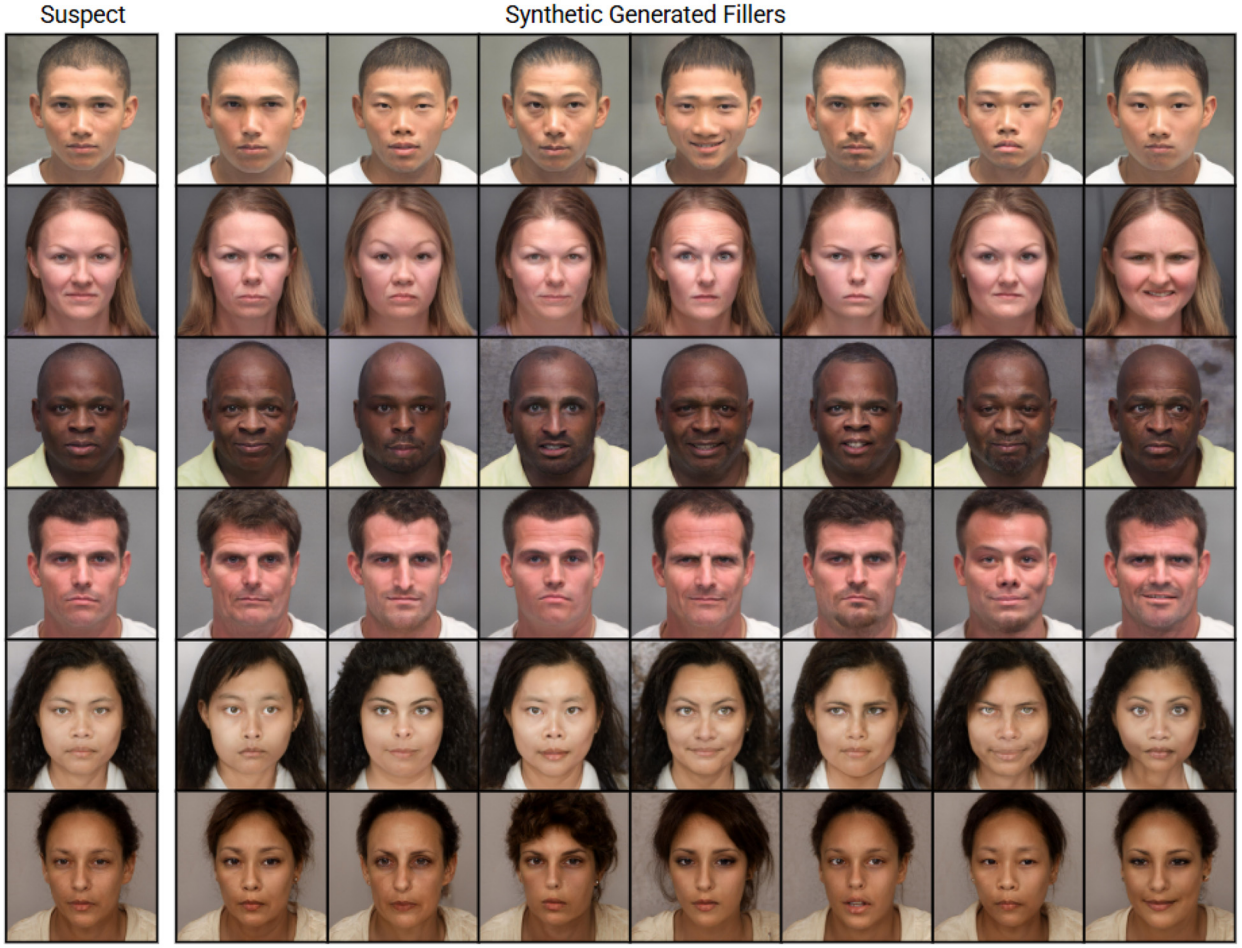
Example of synthetically generated lineup fillers across different demographic groups. Each row shows an inverted suspectimage (left) from the UNCW-MORPH dataset (reproduced with permission from the University of North Carolina Wilmington, Ricanek and Tesafaye, 2006), followed by seven synthetically generated fillers matching the suspect's demographic characteristics. The generation process can be fine-tuned by a noise regulation parameter (epsilon) to adjust facial similarity between the suspect and the fillers.

The aforementioned challenges of finding suitable fillers in a timely and reliable manner across different demographic groups has led to growing interest in alternative solutions to improve lineup fairness and efficiency. While the traditional live lineups offer limited room for innovations, digital medium formats such as photo lineups can benefit from face synthesis techniques (e.g., for the generation of synthetic filler mug shots), which have proven successful in human perception tasks (Joshi et al., 2024; Shen et al., 2021; Nightingale and Farid, 2022). In addition to advantages such as privacy-preservation and compliance with regulatory policies (Parliament and of the European Union, 2016), the generation of synthetic fillers allows for manipulation of facial traits (e.g., identity cues), while simultaneously preserving demographic characteristics, posing a promising alternative to traditional filler selection for lineups. A visual example of synthetic generated fillers by our proposed approach can be seen in Figure 1.

In this work, we propose a practical approach for generating synthetic fillers for use in photo lineups. Our approach allows for efficient filler generation (targeted vector variations) by manipulating a suspect's original latent vector. The degree of facial similarity between a suspect and the generated fillers is controlled through noise regulation. Specifically, a suspect's facial image is inverted into a latent vector representation using the e4e-encoder (Tov et al., 2021), which embeds face images into a structured latent representation within the extended $W^{+}$ latent space of StyleGAN2 (Karras et al., 2020). Synthetic fillers are then generated by injecting weighted random noise into pre-selected layers of a suspect's latent vector representation, such that primarily identity-related traits are altered, while demographic characteristics and face image properties are largely preserved. Additionally, we conducted several experiments, including a large-scale human perception study to investigate the feasibility of synthetically generated fillers for use in photo lineups. Our work contributes to algorithmic approaches improving the fairness of lineups and opens avenues for supporting criminal investigations where synthetic data can be leveraged.

In summary, this work makes the following key contributions:

- We propose a practical approach for the generation of synthetic photo lineup fillers through controlled StyleGAN latent vector manipulations.
- We demonstrate how generative models can support digital crime investigations by enabling efficient and privacy-preserving filler generation, which is particularly useful when the investigation team lacks suitable fillers.
- We provide an empirical evaluation of demographic preservations (age, gender, ethnicity), demonstrating improved preservation performance compared to prior work.
- We conduct a large-scale human perception study with over 450 participants, showing that lineup procedures exclusively consisting of synthetically generated fillers support fair identification performance across demographic groups and participant expertise.

## 2 Related work

The use of generative models in photo lineup procedures (especially for the generation of synthetic fillers) is widely understudied, hence related work remains limited. Nevertheless, there is existing research that is either: (1) using methods for synthetic description-based filler generation, or (2) exploring the (extended) intermediate latent space of StyleGAN2 for appearance-based filler generation.

### 2.1 Description-based filler generation

Greenspan and Bergold (2025) investigated the impact of identification outcomes and human perception across two studies using synthetic filler photos for police lineups. The authors employed a commercial software tool^1^ for the generation of fillers, in which facial traits can be selected to achieve a description-based filler selection. The author's findings indicated no drawbacks to using synthetic filler photos compared to real ones, however the generalizability of the results is limited, as the study focused on white male targets. A comparable finding was reported by Bell et al. (2024): lineups including synthetic fillers resulted in fair and even less biased identifications than lineups containing real filler photos from existing databases. Similar to Greenspan and Bergold (2025), the generation of synthetic fillers in this study was performed using commercial software [Adobe Photoshop 24.7 (Beta)] (Adobe Inc., San Jose, CA, USA), which only allows for the generation of candidates in limited variety (based on the capabilities of the respective software used). Another recent work by Tomašević et al. (2026) introduced Diff-FIT, a diffusion-based framework intended to generate facial compositions based on textual eyewitness descriptions.

### 2.2 Appearance-based filler generation

Other research has explored the (extended) intermediate latent space of StyleGAN2 as a basis for synthetic filler generation. In this domain, a facial image can be inverted into a 512-dimensional latent vector representation w∈W (often referred to as style code or style vector), which can be interpreted by the generator *G* for image reconstruction (Tov et al., 2021). However, Abdal et al. (2019) demonstrated that inverting a given input image into the latent space *W* may not yield reliable reconstruction results and therefore propose to perform inversions within the extended latent space *W*^+^. Consequently, in StyleGAN's extended latent space *W*^+^, an inverted input image is represented by a concatenated layer-wise (18 layers × 512-dimensional) latent vector representation (Abdal et al., 2019). Dokoupil and Peska (2021) introduced LiGAN, a framework for generating synthetic fillers for use in photo lineups utilizing the aforementioned (extended) intermediate latent space. LiGAN applies principal component analysis (PCA) to compress a suspect's style vector and samples random vectors around it (within a hyperball) to generate fillers with similar facial characteristics. However, as sampling is performed around a suspect's PCA-reduced style vector, the encoder's reconstruction fidelity may be limited, while fine tuning involves the risk of mode collapse. Moreover, this approach lacks the capability of control over appearance-based similarity between a suspect and the generated fillers. Another recent work, leveraging the (extended) intermediate latent space of StyleGAN2 is presented by Krzysztof et al. (2025). The authors sample fillers from the neighboring margin region of a suspect's latent vector to ensure similar demographic characteristics, while controlling appearance-based similarity. However, as their proposed filler sampling algorithm involves iterative (gradient descent) optimization via (ILO) Daras et al., (2021), this results in a computational demanding procedure (for each individual suspect) as well as additional drawbacks. In particular, it cannot be ruled out that the filler sampling procedure is repeatedly re-initialized due to margin region *M* and similarity δ constraints, potentially leading to the risk of “getting stuck” in the selection loop without finding a suitable filler. In addition, the authors demonstrated, that the ethnic preservation rate of their generated fillers is unreliable for Asian and Black individuals, resulting in biased lineups (provided that no further post-filtering is performed).

## 3 Methodology

Previous work, such as Wu et al. (2021) or Shen et al. (2020), has demonstrated that StyleGAN's latent space can be utilized to manipulate specific facial traits along trained decision boundaries (e.g., young ↔ old, happy ↔ sad, open eyes ↔ closed eyes). While successful for editing specific visual traits, this approach is rather unsuitable for lineup filler generation. This is because a person's identity is not defined by a single trait (e.g., altering a person's nose), but rather relies on an unknown number of identity cues with varying levels of sensitivity. Consequently, alternative approaches are required to modify a person's identity cues while preserving demographic characteristics.

As outlined in Section 2.2, a face image can be represented in StyleGAN's extended intermediate latent space by a vector representation, with different layers encoding different levels of detail (Melnik et al., 2024). While early layers tend to capture coarse structural traits such as a person's pose, later layers tend to influence fine-grained textures (Shen et al., 2020). Since we aim to alter identity-related cues without changing a suspect's demographic characteristics (age, gender, ethnicity) or image properties (background, lightning, quality), we focus on exclusively manipulating intermediate layers (4–7) of a suspect's laten vector.

The remainder of this section is structured as follows: Section 3.1 introduces a baseline approach that applies layer-wise random noise injection to generate vector variations (fillers). Following, Section 3.2 presents a sensitivity evaluation of how injected noise strength influences facial similarity and face image quality across latent layers. Finally, Section 3.3 describes our extended weighted-noise injection method based on the findings of the sensitivity evaluation.

### 3.1 Baseline approach: layer-wise noise injection

This section describes our baseline approach, to generate synthetic lineup fillers by injecting random noise into latent layers of a suspect's vector representation. As we exclusively inject random noise into selected intermediate layers (4-7), we assume that these stochastic vector variations cause identity-related changes, while largely preserving demographic characteristics and image properties.

Firstly, we invert a suspect's facial image (via the e4e-encoder Tov et al., 2021) into a latent vector representation **w^+^**. For each latent layer *l*∈{4, 5, 6, 7}, we manipulate the original latent vector representation by injecting random noise drawn from a normal distribution N(0,I). We restricted noise injection to these intermediate layers, as prior exploration of StyleGAN's latent space have shown that early layers primarily encode coarse structural traits, while later layers tend to control fine-grained textures (Shen et al., 2020). We therefore assume that the intermediate layers (4–7) most strongly encode identity-related traits, while demographic characteristics (age, gender, ethnicity) are mainly preserved. The random noise is normalized and scaled using a noise regulation parameter ε (regulation the strength of facial trait variation) and applied to the corresponding layers. Our proposed baseline approach is summarized in Algorithm 1.

Algorithm 1 Firstly, a suspect's face image is inverted [via the e4e-encoder (Tov et al., 2021)] into a latent vector representation **w**^+^. For each latent layer *l*∈{4, 5, 6, 7}, normalized Gaussian noise scaled by ϵ to the suspect's latent vector *w*^+^. 
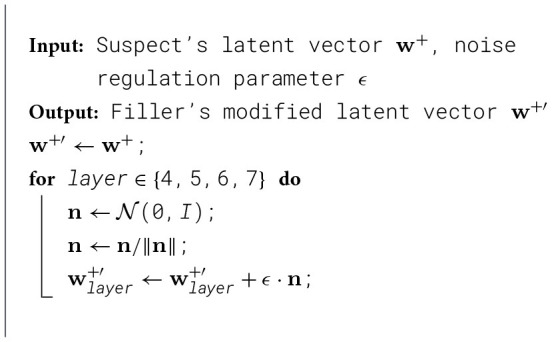

The manipulated vector variation *w*^+′^ is then decoded to reconstruct the filler image.

### 3.2 Noise sensitivity analysis of latent layers

Since we assume that the noise injected into a suspect's original latent vector representation will have varying degrees of impact (layer-wise), we conducted extensive experiments to determine the sensitivity of a suspect's latent vector layers (4–7) to changes in the added noise intensity regulated by Epsilon. In particular, we investigated how the noise intensity regulated by Epsilon affects facial features in the encoded face representation and subsequently variations in facial similarity, while also analysing how face image quality is affected by varying levels of injected noise strength. The parallel assessment of face image quality at varying levels of epsilon noise is an important metric, as we want to ensure that the synthetically generated fillers not only match a suspect's appearance to a certain extent, but also do not exhibit any significant image deficiencies.

For the experimental setup we compiled a subset of the HDA-SynChildFaces database (Falkenberg et al., 2024). We choose HDA-SynChildFaces, as it represents a demographically balanced large-scale face image database, in which facial images have been synthetically generated via StyleGAN3 (Karras et al., 2021). For determining accurate Epsilon noise sensitivity, the origin of these face images (StyleGAN) is additionally beneficial, as many GAN encoders (such as the utilized e4e encoder Tov et al., 2021, which inverts a facial image into the latent vector, that reconstruct the original facial image most accurately) are pre-trained on similar image distributions (e.g., FFHQ), contributing to more accurate and distortion-free latent vector representations. Facial images included in our compiled subset were limited to adult reference images and further processed via the Open Source Face Image Quality (OFIQ)^2^ software to filter out face images with an unified quality score (UQS) below the 25th percentile. This UQS estimates the overall (unified) face image quality with respect to any potential image deficiency and can be used to ensure that facial images that have not been assigned a high UQS (facial images of insufficient quality for police lineups) are excluded. Additionally, we balanced the remaining face images across various ethnicities to ensure equal representation of demographic variations.

For each remaining facial image (simulated suspects) in the compiled subset, we generated 25 latent vector variations (simulated fillers) over a broad Epsilon noise interval [5,75]. This procedure was conducted for each analyzed vector layer individually, resulting in 24,900 synthetically generated fillers per altered layer (996 original suspects × 25 Epsilon variations). Given the demographic diversity and the number of generated fillers, reliable conclusions can be drawn about how the choice of Epsilon affects facial similarity and face image quality layer-wise.

**Figure 2:**
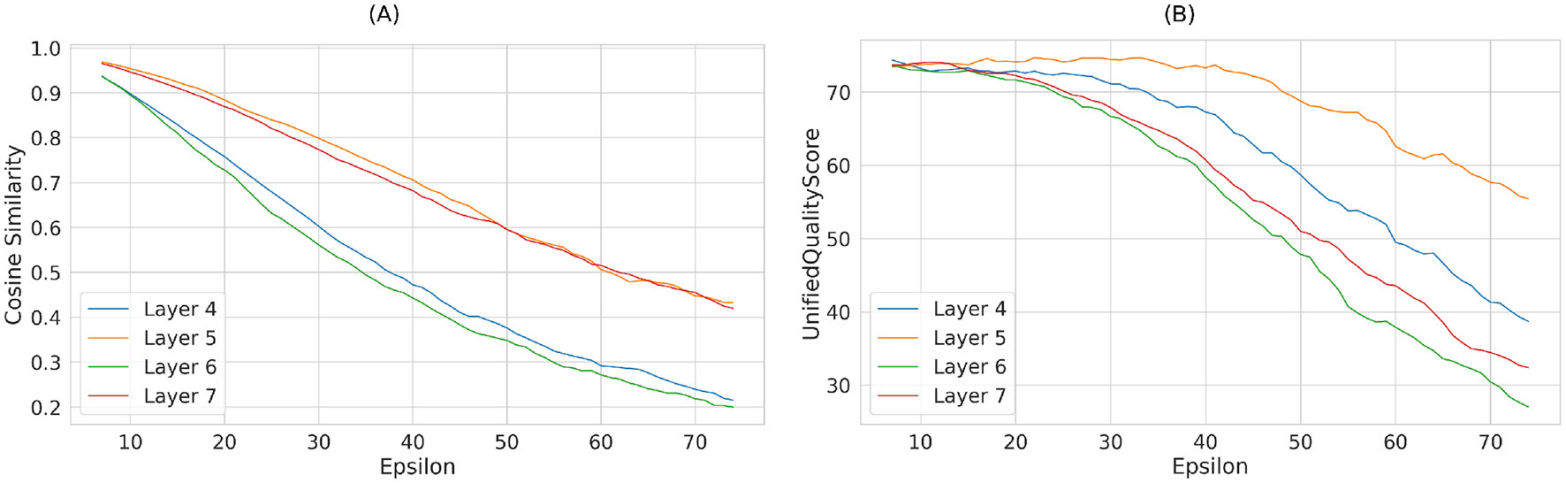
Comparison of smoothed mean cosine similarity **(A)** and smoothed mean UQS **(B)** across latent vector layers and epsilon noise-strength values. Higher epsilon values (*x*-axis) correspond to stronger noise perturbations applied during filler generation. Visualizations were smoothed using a centred moving window (window size = 4).

To measure the degree of appearance-based similarity between a suspect and the corresponding generated fillers, we extracted face embeddings via MagFace (Meng et al., 2021). Subsequently, for each e4e-inverted suspect image, the cosine similarity between its MagFace embedding and the 25 corresponding filler embeddings was compared. Additionally, the effect of variation in epsilon on face image quality (unified) was investigated, as demonstrated in Figure 2A for facial similarity and Figure 2B for face image quality respectively. For visualization and trend analysis, we grouped the results by calculating the mean cosine similarity and mean UQS per epsilon strength. To better highlight overall trends (smoothing) in the visualization, we applied a centered moving window with a window size of four epsilon values.

Taking a closer look at Figure 2A, the following trends can be observed: the effect of increasing noise strength on the facial features present in the latent vector representation on layers 5 and 7 has, on average, less influence on appearance-based face similarity (cosine similarity) than the facial features represented in layers 4 and 6. Notably, layers 4 and 6 as well as layers 5 and 7 are particularly close to each other, suggesting that the facial features they contain respond with similar sensitivity to changes in the noise strength regulated by epsilon. This presumably suggests that the facial features in layers 4 and 6 tend to include more identity cues (as these have a stronger influence on facial similarity) rather than features responsible for semantic changes such as, e.g., the facial pose (which is assumed to have less influence on changes in cosine similarity). Looking at Figure Figure 2B, it is noticeable that as the epsilon values increase (increased noise strength), the face image quality is affected to varying degrees depending on the layer. It is noticeable, that layer 5 maintains a similar face image quality (UQS) over a longer epsilon interval, while layer 6 shows the greatest decline in face image quality (UQS) when applying higher epsilon values. Therefore, it is necessary to consider these UQS trends when generating fillers, as the UQS should not be too low and, accordingly, the epsilon value, responsible for the injected noise strength should not be selected too high.

### 3.3 Weighted layer-wise noise injection

**Figure 3:**
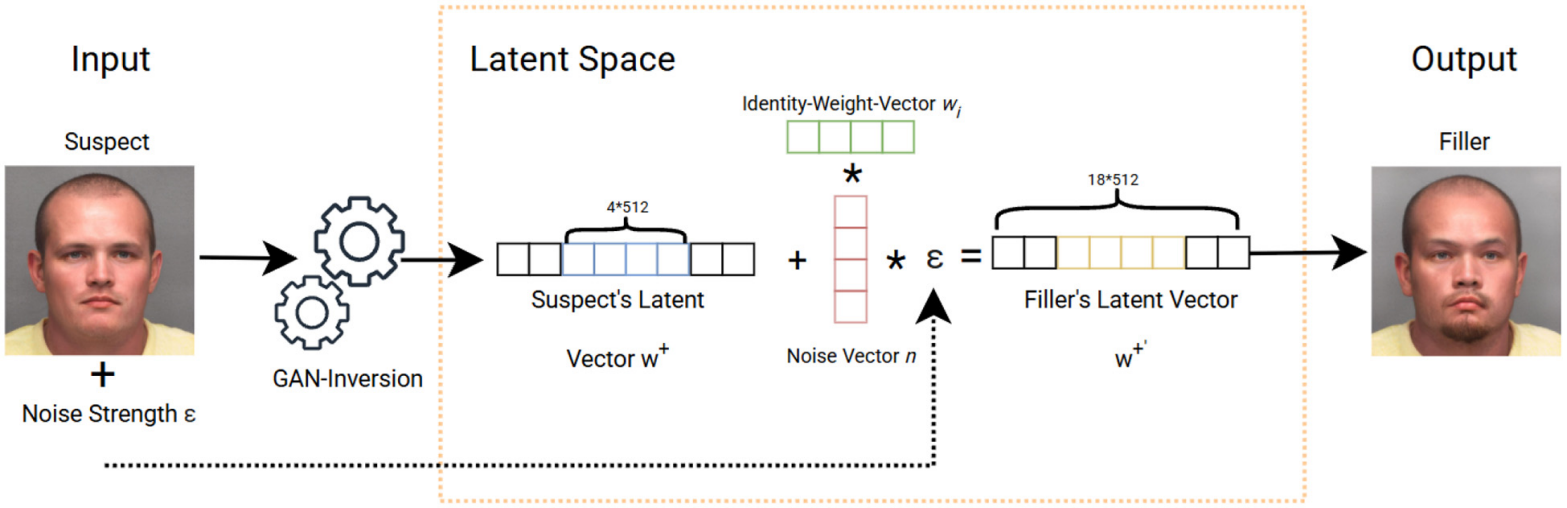
High-level overview of the weighted layer-wise noise injection approach (input image reproduced with permission from the University of North Carolina Wilmington, Ricanek and Tesafaye, 2006). A suspects facial image is inverted into a latent vector representation, which is manipulated by injecting random noise in selected intermediate layers. The resulting filler image can then be reconstructed using the manipulated latent vector.

As illustrated in Figure 3, our approach takes a suspect's facial image as input and inverts it into a latent vector representation **w^+^** using the e4e-encoder (Tov et al., 2021). In addition to a suspect's facial image, a Epsilon ε (noise-strength regulation value) is expected. A smaller ε results in more subtle visual changes, whereas a larger ε leads to greater visual dissimilarities between a suspect and the generated filler. Following, the suspect's original latent vector representation is manipulated by injecting random noise drawn from a normal distribution N(0,I) for each layer in *layer*∈{4, 5, 6, 7}. The random noise is scaled using the noise regulation parameter ε, multiplied by pre-computed layer-wise identity weights, and applied to the corresponding layers. The filler mug shot is then reconstructed using the targeted latent vector variation **w^+^′**.

As we want to give greater consideration to identity cues than to other (unknown) factors (e.g., the head pose) potentially present in a suspect's latent vector layers (4–7), we adopt an extended layer-wise weighted noise injection approach based on the experimental results visualized in Figure 2. In this extended approach, each latent vector layer (4–7) is weighted more or less strongly depending on its influence on the appearance-based (cosine) face similarity. In particular, we calculated the area under the curve (AUC) for the cosine similarity trends shown in Figure 2A. Lower AUC values indicate greater sensitivity of a layer to identity-related changes, as stronger decreases in cosine-similarity correspond to larger appearance-based deviations from the original suspect. In particular, inverse AUC values

$${ŵ}_{i}=\frac{1}{{\text{AUC}}_{i}}$$

were calculated and subsequently normalized to derive the corresponding layer-specific identity weights. The noise scaling parameter ε is then multiplied by the corresponding calculated identity-weight

$$w_{i}\in \left\{1.62,0.20,1.93,0.26\right\}$$

so that layers with stronger identity cues (layer 4 and 6) receive larger noise perturbations than layers 5 and 7. This refers to extending the presented Algorithm 1.

$$w^{+}\prime_{layer}\leftarrow w^{+}\prime_{layer}+\varepsilon \;\cdot \;n$$

with

$$w^{+}\prime_{layer}\leftarrow w^{+}\prime_{layer}+\varepsilon \;\cdot \;n$$

where *w*_*i*_ denotes the precomputed layer-specific identity weight. A visual demonstration of our proposed system can be seen in Figure 3.

**Figure 4:**
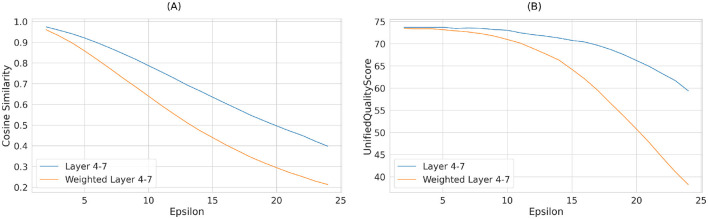
Comparison of smoothed mean cosine similarity **(A)** and smoothed mean UQS **(B)** across epsilon noise-strength values.Higher epsilon values (*x*-axis) correspond to stronger noise perturbations applied during filler generation. The orange line visualizes the approach in which layers get weighted by their impact on cosine-similarity. Visualizations were smoothed using a centred moving window (window size = 4).

We then compared this layer-specific weighted noise injection approach with the preliminary (non-weighted) injection approach described in Algorithm 1 to determine the sensitivity to changes in appearances-based similarity and face image quality by Epsilon. To this end, we generated 25 filler variants (24,900 fillers in total per algorithm approach) for a suspect and grouped the results for visualization and trend analysis by calculating the mean cosine similarity and the mean UQS per epsilon strength, as we previously did for the layer-wise analysis. The results are visualized in Figure 4A for facial similarity and Figure 4B for face image quality respectively.

Taking a closer look at Figure 4A, one can notice that the extended approach (including layer-wise identity weighting) is more sensitive to changes in the noise strength Epsilon than the native approach (without layer-wise identity weighting). This behavior is to be expected, as layers containing stronger identity cues (layer 4 and 6) receive larger noise perturbations, leading to a stronger deviation in appearance-based similarity. Additionally, increasing epsilon values result in a progressive degradation of face image quality (UQS) on average, as demonstrated in Figure 4B. Therefore, choosing excessively high epsilon values should be avoided, as they negatively affect face image quality by introducing strong visual artifacts and implausible facial appearance in the generated fillers.

### 3.4 Summary of the filler generation approach

Our proposed method aims to support criminal investigations by generating suitable synthetic lineup fillers. This approach is particularly useful when the investigation team lacks suitable fillers and synthetic data can be leveraged. Synthetic lineup fillers are generated by targeted manipulation of a suspects original latent vector representation. The approach presented in Section 3 comprises the following steps and can be summarized as follows:

1. **Latent inversion**: a suspect mugshot is first inverted into its latent vector representation using the e4e-encoder (Tov et al., 2021).
2. **Layer-wise noise injection**: random noise drawn from a normal distribution N(0,I) is injected into the intermediate latent layers *l*∈{4, 5, 6, 7} to introduce controlled variations in identity-related facial cues. Noise strength is normalized and scaled using a noise regulation parameter ε.
3. **Identity-weighted noise scaling**: the injected noise is scaled by layer-specific identity-weights *w*_*i*_∈{1.62, 0.20, 1.93, 0.26} derived from the sensitivity experiments of cosine-similarity trends (see Section 3.2).
4. **Image reconstruction**: the manipulated latent vector is decoded by the generator to reconstruct the synthetic filler mugshot.

## 4 Experiments

### 4.1 Preservation of demographic characteristics

To demonstrate that our synthetically generated fillers are capable of sufficiently matching the demographic characteristics (age, gender, ethnicity) of associated suspects while changing facial traits, we performed a demographic-preservation experiment. This experiment is highly relevant because it (1) provides insights into the reliability of our approach and (2) investigates whether there are preservation differences across demographic groups that could lead to potentially biased lineups. To compare the demographic preservation rates of the generated fillers with recent work, we utilized the same evaluation dataset (simulating lineup suspects) used in Krzysztof et al. (2025). As described in (Krzysztof et al. 2025), this evaluation dataset is a subset comprising 300 authentic (manually selected) mug shot photographs equally distributed across age and ethnicity, from the UNCW-MORPH dataset (Ricanek and Tesafaye, 2006). This database is particularly well-suited for such an analysis because it contains authentic mug shots, thus simulating a real scenario for potential suspects and filler selection.

In this experiment, for each individual filler generation process, we randomly selected the layer noise intensity from a discrete uniform distribution over the Epsilon interval [8,10]. For this experimental setup, we chose this epsilon interval based on the results from Figure 4. This allows us to achieve a high average UQS and at the same time ensure sufficient cosine similarity per generated filler. However, due to the behavior of our presented approach, it should be noted that we cannot guarantee an exact desired facial similarity between a suspect and the generated fillers through controlled noise scheduling and outliers can yet occur. To ensure the same experimental lineup size (number of lineup candidates) as in Krzysztof et al. (2025), seven filler photos were created per suspect, as suggested by a decree from the North-Rhine Westphalian Ministry of the Interior (Ministry of the Interior Nordrhein-Westfalen, 2006). This degree is consistent with current research-based police lineup recommendations suggesting at least five fillers per lineup (Smalarzand and Wells, 2024).

As in Krzysztof et al. (2025), we utilized a commercial off-the-shelf (COTS) software to estimate the demographic characteristics (age, gender, ethnicity) of the generated fillers. We note that such predictions, like any other demographic estimation tool, may be subject to classification errors or underlying biases. These predictions were then compared against the suspects ground-truth demographic characteristics. Overall, our experimental results show promising preservation rates across all observed demographic characteristics. Particularly notable is the significant improvement in the total ethnicity preservation rate by 40% compared to the prior approach in Krzysztof et al. (2025), as shown in Table 1. This result is primarily influenced by the improved preservation rate of the Black (+61,3%) and Asian (+23,9%) ethnicity groups. However, these two ethnicity groups still demonstrate larger discrepancies compared to the preservation rate for White individuals. Especially when conducting a photo lineup with an Asian suspects, synthetically generated filler candidates may have different ethnic origins. As shown in Table 2, our approach results in slightly improved age preservation (with a 1,3 year lower MAE) compared to the method presented in Krzysztof et al. (2025). Overall the age preservation rates appear consistent with only minor deviations across the demographic groups. Table 3 presents the gender preservation rate between suspects and their matched fillers. The overall gender preservation rate remains relatively consistent across the two approaches. However, Asian males show an increased gender preservation rate of 12,8%, while the preservation rate of Black females shows a decrease of 13,3%.

**Table 1:** Ethnicity preservation rate (in %) between suspects and fillers.

Method	Category	Asian	White	Black	Total
Identity-Parade (Krzysztof et al., 2025)	Female	25.4	99.7	17.2	47.9
	Male	32.8	100.0	17.9	50.6
	Total	29.1	99.9	17.6	49.4
Ours	Female	54.6	97.1	76.3	89.7
	Male	51.4	99.0	81.4	89.0
	Total	53.0	98.1	78.9	**89.4**

Bold values highlight the better overall (total) performance between the compared methods.

**Table 2:** Age mean absolute error (MAE) between suspects and fillers.

Method	Category	Asian	White	Black	Total
Identity-Parade (Krzysztof et al., 2025)	Female	9.7	8.7	10.4	9.6
	Male	6.0	8.3	8.0	7.5
	Total	7.9	8.5	9.2	8.5
Ours	Female	8.8	6.4	8.5	7.9
	Male	5.6	6.9	6.8	6.4
	Total	7.2	6.7	7.7	**7.2**

Bold values highlight the better overall (total) performance between the compared methods.

**Table 3:** Gender preservation rate (in %) between suspects and fillers.

Method	Category	Asian	White	Black	Total
Identity-Parade (Krzysztof et al., 2025)	Female	99.6	94.6	89.6	94.2
	Male	66.3	84.4	78.8	76.6
	Total	82.7	89.5	84.2	85.5
Ours	Female	94.0	89.4	76.3	86.6
	Male	79.1	89.4	81.4	83.3
	Total	86.6	89.4	79.0	85.0

Bold values highlight the better overall (total) performance between the compared methods.

It should be noted that the preservation deviations across the demographic groups may additionally be influenced by various sources, however an in-depth bias investigation is beyond the scope of this work. One source of influence, mentioned in (Krzysztof et al. 2025), are existing biases in the StyleGAN2 generator Karras et al., (2019), potentially caused by the underlying demographically unbalanced FFHQ dataset Maluleke et al., (2022).

In addition, the COTS predictions of a fillers demographic characteristics may introduce mismatches between the ground-truth labels of a suspect and the estimated fillers characteristic, potentially leading to erroneously reduced preservation rates in certain cases. For example, when a filler is generated to match the associated suspects ethnicity (e.g., White), the COTS estimation might classify a suspects ground-truth label differently (e.g., Asian), resulting in labeling differences rather than visual dissimilarities.

### 4.2 Human perception study

We conducted an extensive human perception study to evaluate the suitability of our synthetically generated fillers for use in photo lineups. For conducting the study, we utilized the web-based *Unclassifyd* framework, previously introduced in Nichols et al. (2022) for psychophysical evaluation of human performance. The study investigates how synthetically generated fillers are perceived by human observers, focusing on suspect classification rates, self-reported decision confidence, and classification duration.

#### 4.2.1 Participant recruitment

In total, 452 valid participations (52.4% male, 46.5% female, and 1.1% non-binary or prefer not to say) were recorded.^3^ A participation was considered valid if a participant (1) completed all 6 lineups, (2) selected one face image per lineup, and (3) reported their decision confidence on a scale from 0 to 100, where higher values indicate greater certainty (with which the participant is convinced they have selected the correct suspect). This resulted in 2,712 lineup classifications (six per participant). The study distinguished between 44 biometrics experts (recruited via academic research groups and domain networks), and 408 non-experts (recruited via the Prolific platform^4^).

#### 4.2.2 Experimental procedure

The task of each participant was to identify a suspect from a photo lineup consisting exclusively of synthetically generated fillers. For each demographic combination of ethnicity (Asian, Black, White) and gender (Male, Female), we selected one mug shot from the UNCW evaluation dataset as the suspect. Using our proposed approach, we generated seven lineup fillers per demographic group to comply with the decree from the North-Rhine Westphalian Ministry of the Interior. Filler candidates for the human perception study were generated using moderate epsilon noise-strength values with ϵ∈[10, 14]. The generation process followed an iterative human-in-the-loop procedure, in which batches of approximately 50 candidates per suspect were generated and visually analyzed. Candidates exhibiting visible artifacts or implausible facial appearance introduced by the latent vector perturbations were discarded and additional candidates were generated as needed. The final seven fillers per demographic group were then manually post-filtered based on the aforementioned criteria.This procedure is intended to reflect the operational use of our proposed approach, where synthetic fillers are visually inspected and verified by human operators, as lineups remain a sensitive decision-making process in which human observation is essential.

**Figure 5:**
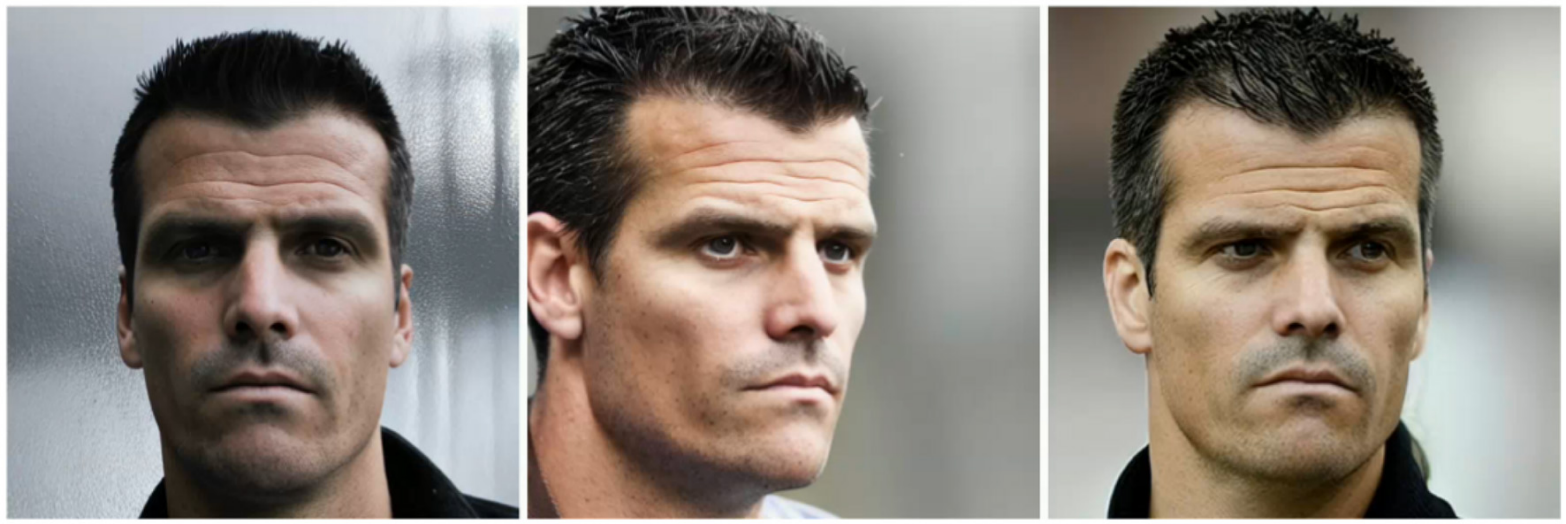
Suspect exposure phase: before the line up identification, participants were shown three synthetically generated “in-the-wild” mated face images of the suspect for 10s.The images were generated via Arc2Face (Papantoniou et al., 2024) based on the corresponding UNCW mugshot (input image reproduced with permission from the University of North Carolina Wilmington, Ricanek and Tesafaye, 2006).

To simulate an operational eyewitness scenario, participants were first shown three synthetically generated “in-the-wild” face images (mated samples) of the corresponding UNCW mug shot for 10 s^5^, as illustrated in Figure 5.

**Figure 6:**
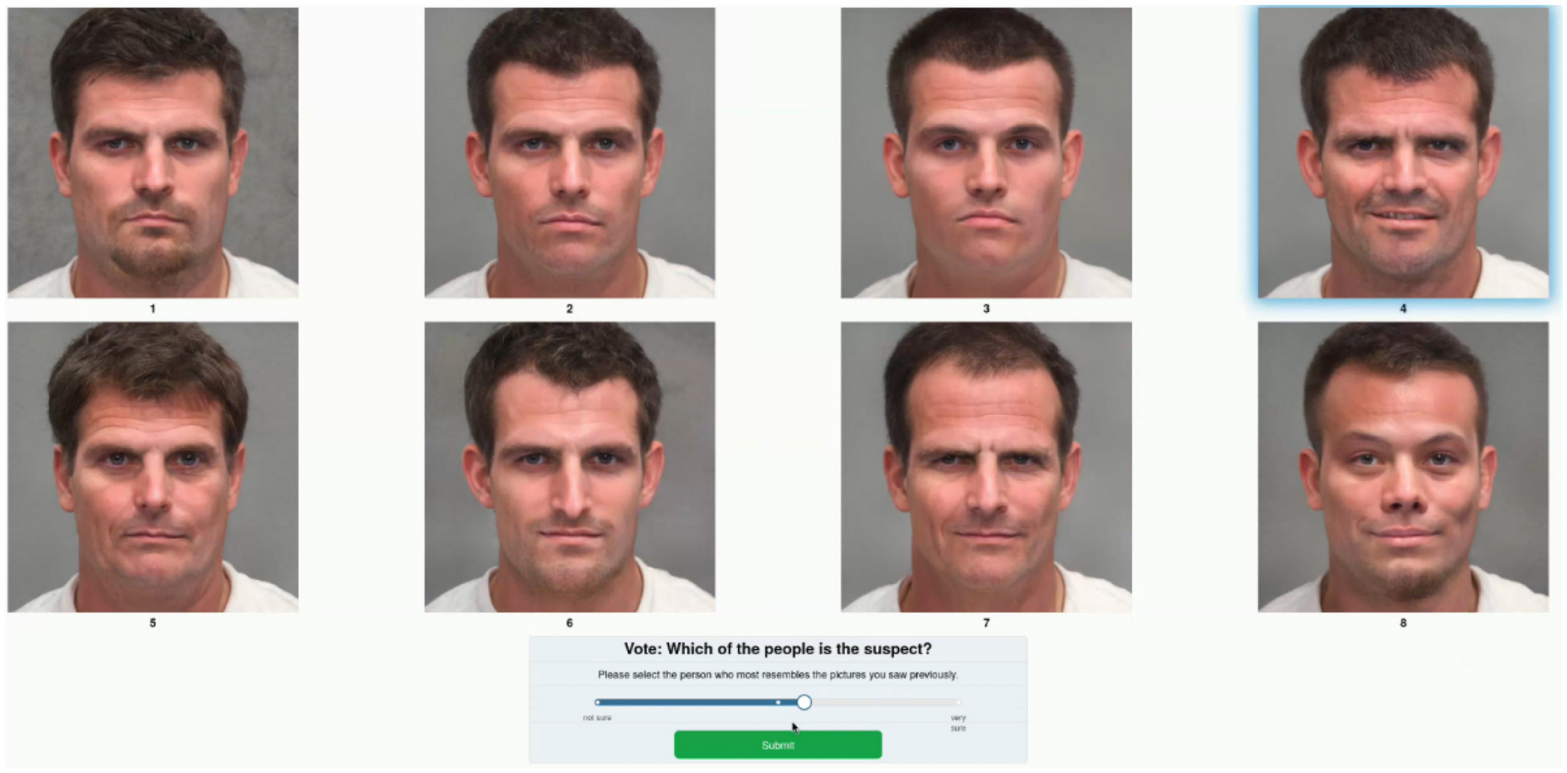
Lineup identification phase: participants were asked to identify the previously seen suspect from a lineup of eight facial images (input images reproduced with permission from the University of North Carolina Wilmington, Ricanek and Tesafaye, 2006). The selected photo (blue outline) corresponds to a misclassification (the correct suspect is number 2). The bar below the lineup indicates the participants self-reported decision confidence.

After viewing the “in-the-wild” images, participants were distracted with a brief cognitive task^6^ to reduce short-term memory effects. The task could be stopped after 60 s, after which the corresponding lineup was presented. Figure 6 visualizes the corresponding identification phase for the previously depicted suspect exposure phase. To reduce lineup arrangement biases, the order of the displayed face images within the lineup was randomized per participant. In addition, the order of the six lineup procedures was randomized per participant. The set of displayed face images remained identical for all participants.

#### 4.2.3 Evaluation metrics and analysis

We analyzed the suspect classification rate as a key metric reporting the proportion of correctly identified suspects. In addition, we analyzed a participants identification phase duration^7^ and self-reported decision confidence as indicators of lineup difficulty and cognitive effort. Table 4 reports the suspect classification rates per lineup demographics across participant expertise. High classification rates would suggest trivial lineups in which the suspect stands out due to insufficient similarity to the fillers. Conversely, low suspect classification rates would suggest overly difficult lineups, presumably due to strong visual similarities between the suspect and the fillers.

**Table 4:** Suspect classification rates separated by participants expertise.

	Biometric-experts (%)				Non-experts (%)			
Lineup	Asian	Black	White	Total	Asian	Black	White	Total
Female	81.8	61.4	56.8	66.7	82.1	67.2	56.1	68.5
Male	43.2	90.9	77.3	70.5	53.2	83.1	78.7	71.7
Total	62.5	76.2	67.1	68.6	67.7	75.2	67.4	70.1

Gender and ethnicity correspond to the lineup procedure and are independent of the participants demographics.

The results indicate that the proposed synthetic fillers enable fair and balanced lineups across demographic groups. Biometric experts achieved an overall suspect classification rate of 68.6%, compared to 70.1% for non-expert participants. These rates suggests, that the fillers used neither trivialize the identification process (the suspect does not stand out) nor lead to extremely difficult lineups with suspect classification rates at random chance (12.5% in an identification setup of eight individuals). Of the 452 valid participants, 77 (17%) correctly identified all six suspects, suggesting that all conducted lineup procedures are solvable. Biometric experts reported an average decision confidence of 63.6% and required on average 26.1 s per identification phase. Non-expert participants reported an average decision confidence of 69.3% and required on average 22.7 s per identification phase. Across the demographic groups, variations in suspect classification rate can be observed: higher suspect classification rates (e.g., for black male) may suggest that the corresponding suspect exhibit more distinctive (visual) identity cues relative to the fillers, whereas lower suspect classification rates (e.g., for Asian male) may indicate a higher degree of visual similarity between the suspect and the generated fillers.

**Figure 7:**
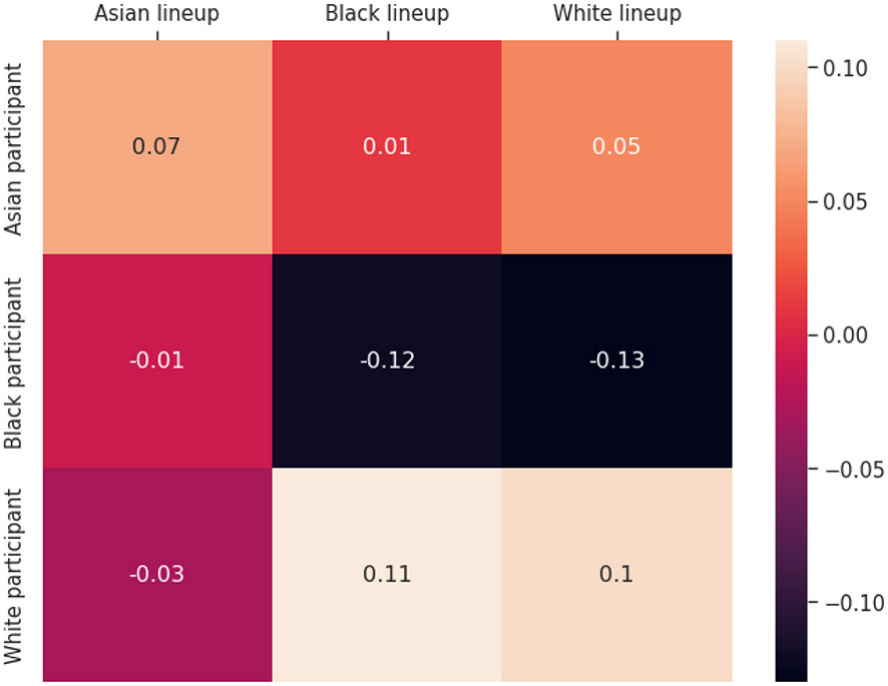
Correlation between participants ethnicity and suspect classification performance per lineup.

However, suspect classification rates may also be influenced by various factors related to human perception, such as the Other-Race Effect (Stelter and Schweinberger, 2023; Furl et al., 2002) (ORE), where individuals tend to recognize faces belonging to their own ethnic group more accurately compared to those from other ethnicities. To further investigate potential influences of human perception on lineup decision-making, we analyzed the correlation between participants ethnicity and suspect classification performance. In this analysis, the suspect classification rate was calculated per participant rather than per lineup trial. Figure 7 reports the resulting correlations between participant ethnicity and lineup accuracy for each demographic group.

The observed correlations indicate small differences in identification performance across participant ethnicities. While weak positive correlations can be observed for same-ethnicity scenarios (e.g., white participants identifying white lineups or Asian participants identifying Asian lineups), the strongest negative correlation can be observed for black participants identifying white lineups, which might be influenced by factors related to human perception such as the ORE. In this experimental setup, black participants tend to achieve lower identification performance compared to participants from other ethnic groups. These findings should be treated cautiously due to the limited number of lineups per participant and the use of identical lineup sets across participants. However, observed effects might be more prominent in operational scenarios with greater lineup variability. Overall, none of the lineups resulted in near-perfect or near-random identification. These results support the use of generated fillers in photo lineups, particularly when the availability of suitable fillers is limited. However, given the variations in witness identification caused by various factors of human perception (e.g., the ORE), ethnically diverse pairings (ethnicity of the witness ≠ ethnicity of the lineup candidates) should be handled with caution.

## 5 Limitations

The human perception study followed a target-present identification design based on six lineup trials per participant. Although the order of displayed lineup images was randomized per participant, the study was limited to a fixed set of displayed lineup faces (including the suspect) across all participants. Consequently, false-identification rates for target-absent lineup procedures are not estimated. We note, that demographic correlation effects observed in Figure 7 are based on identical lineup sets per participant and may introduce identity-specific correlations, which can derive from operational scenarios with greater lineup variability.

Although the proposed filler generation approach shows promising potential for supporting digital criminal investigations, as confirmed by the results of the conducted human perception study, a key limitation is that large manipulations of a suspects latent vector representation (caused by excessively large noise scaling) degrade visual appearances in the resulting filler (see Section 3.2). We therefore recommend incorporating a post-processing step, allowing investigation teams to manually select and verify appropriate candidates from a pool of generated fillers and discard those exhibiting visual artifacts. Furthermore, we would like to point out that the proposed method is intended as a tool to assist with digital investigations, rather than a fully automated approach without human supervision. This is because, in the context of an investigation, the selection of individuals for a lineup remains a sensitive decision-making process in which human observation is essential. Synthetically generated fillers should therefore be verified by a (police) attendant before being included in a (police) lineup (provided that the use of synthetic data is permitted).

## 6 Conclusion

In this work, we proposed an algorithmic approach for generating synthetic photo lineup fillers. We address practical forensic challenges to mitigate the risk of misidentifications, particularly in real-world scenarios where the investigation team lacks suitable fillers. Our method generates synthetic fillers through targeted vector variations by injecting weighted random noise into a suspects latent vector representation, enabling controlled manipulation of identity-related cues.

We conducted a large scale human-perception study to evaluate the potential of synthetically generated fillers for use in photo lineups. Across all demographic lineup procedures, the synthetic fillers (1) neither trivialize the identification process nor (2) made suspects indistinguishable, demonstrating that synthetic fillers contribute to fair and balanced lineups. Minor deviations in suspect classification rates across demographic pairings (ethnicity particpant ≠ ethnicity lineup candidates) reflect performance effects caused by various factors of human perception, suggesting handling diverse pairings with caution. In addition, experimental results indicate strong preservations of demographic characteristic between suspects and their corresponding fillers, further supporting the reliability of our proposed approach.

Overall, our approach enables the timely and privacy-preserving generation of suitable lineup fillers, supporting real-world scenarios where suitable fillers are limited. Instead of manually generating fillers with traditional photo editing software, investigators can generate, verify and select suitable candidates from a pool of synthetic fillers, providing a practical and scalable alternative to traditional approaches and supporting the arrangement of fairer and more consistent photo lineups in operational settings.

## Data Availability

The human perception data from this study can be made available upon request.

## Footnotes

1
https://generated.photos/

2
https://github.com/BSI-OFIQ/OFIQ-Project

3 Dropouts and incomplete participation were excluded from the evaluation.

4
https://www.prolific.com/

5 For each mug shot, we generated 500 images, calculated the cosine similarity to the mug shot using MagFace, and selected the three generated “in-the-wild” images with the highest similarity scores.

6 Participants played the puzzle game 2048 https://play2048.co/about.

7 The time taken by participants to select a displayed face image from the lineup.
